# The Demands of the Age upon Our Profession

**Published:** 1864-09

**Authors:** J. Taft


DENTAL REGISTER OF THE WEST.

VOL. XVIII.] SEPTEMBER, 1864. [NO. 9.

### Original Essays and Communications.

### THE DEMANDS OF THE AGE UPON OUR PROFESSION.

BY J. TAFT.

In all departments of human thought and effort, in this period of the world's history, a utilitarian spirit stands prominent. There is an almost universal disposition, to make the most of every thing, and turn every thing to the best account. In a very special manner, is the practice of medicine, in all its departments, characterized by a utilitarian spirit. The practice of medicine, either as a whole, or in any of its specialities, must result in good,—in the prevention of human suffering, and the production of happiness, otherwise it would be discarded. Our own speciality, dentistry, must needs partake of this same characteristic, in order to answer the general requirements of the age upon it; and he who practices his profession, with a determination to produce the largest results, in doing good, most nearly, answers the requirements of the age upon him.

Other pursuits and avocations of life, far less intimately connected with the comfort and well-beings of humanity, seem by their own spontaniety to be more and more flowing into utilitarian channels. This common current then, indicates somewhat the requirements of the present period upon our profession.

No one then, who engages in a calling with which the comfort of his fellows is so intimately connected, should make the attainment of gain his highest object. The dentist who practices his profession with no higher aim than pecuniary emolument, should at once abandon it, and seek some other, having less connection with human weal and woe.

We do not say or believe, that any one should go forth to the performance of his work, wholly oblivious to the thought of remuneration, for this, in the nature of things, is made a stimulus to the well doing of that which is performed; but the rendering of comfort and happiness to others should be the ruling thought, because it is a purer, more holy and happifying one, and tends to elevate and make better, while the simple love of money tends to debase, and make one less good and happy.

Again, he who would well accomplish that which he undertakes, should have a love for it. As a rule, no one ever does well that which he dislikes to do, at least it will not be as well done, as though there was joy in the doing.

Again, this is an age of progress; it is a time when he who will not run must be left behind. In almost all departments progression is a watchword; no one can stand still, if he would, unless he shuts his eyes, and anchors himself down with hooks of steel to old ideas and practices.

Occasionally, in our profession, we hear a man say, "I see nothing new;" we have also heard a man say at mid-day, "I do not see the light of the sun," yet it did shine brightly nevertheless.

Perhaps there are two reasons why men do not progress, why they see nothing new and interesting, why they do not see beautiful objects in the bright light about them; some are blind, their eyes covered with a film and scales, through which no light, however, bright can penetrate,—for such an operation for cataract is required.

Others again, though they have some vision, see men as trees walking, and fail to apprehend, and apply that which they know exists; for such the present rapidity of progress is too great, they have not been accustomed to move either mentally or physically, so that new things have passed so swiftly on, that they have caught only the shadow instead of the substance.

Progress is the spirit of the age, in all things, and unless we imbibe the spirit and act upon its teachings, we do not respond to the demands of the age upon us; "old things are passing away, and all things are becoming new," he, who will tarry in the old, must pass away with them.

